# Supplementary material for: Modeling glioblastoma heterogeneity as a dynamic network of cell states
Source: Mol Syst Biol. 2021 Sep 16;17(9):e10105. doi: 10.15252/msb.202010105 (PMC8444284; doi:10.15252/msb.202010105)
Supplement: Supplementary file 5 — Source Data for Figure 3 [file MSB-17-e10105-s001.zip › Figure3A_sourcedata/GSEA_3065/hallmarks_state4.GseaPreranked.1623416296068/HALLMARK_ANDROGEN_RESPONSE.html]

Details for gene set HALLMARK\_ANDROGEN\_RESPONSE[GSEA]

|  || Dataset | state4 |
| Phenotype | NoPhenotypeAvailable |
| Upregulated in class | na\_neg |
| GeneSet | HALLMARK\_ANDROGEN\_RESPONSE |
| Enrichment Score (ES) | -0.48231548 |
| Normalized Enrichment Score (NES) | -1.6219782 |
| Nominal p-value | 0.008720931 |
| FDR q-value | 0.023980204 |
| FWER p-Value | 0.229 |
Table: GSEA Results Summary

  

Fig 1: Enrichment plot: HALLMARK\_ANDROGEN\_RESPONSE      
 Profile of the Running ES Score & Positions of GeneSet Members on the Rank Ordered List

  

| PROBE | GENE SYMBOL | GENE\_TITLE | RANK IN GENE LIST | RANK METRIC SCORE | RUNNING ES | CORE ENRICHMENT || 1 | SAT1 |  |  | 4 | 0.669 | 0.0970 | No |
| 2 | TSC22D1 |  |  | 369 | 0.160 | 0.0618 | No |
| 3 | PLPP1 |  |  | 785 | 0.105 | 0.0102 | No |
| 4 | NCOA4 |  |  | 1176 | 0.077 | -0.0413 | No |
| 5 | ARID5B |  |  | 1327 | 0.070 | -0.0553 | No |
| 6 | APPBP2 |  |  | 1344 | 0.069 | -0.0478 | No |
| 7 | ZBTB10 |  |  | 1490 | 0.062 | -0.0621 | No |
| 8 | RAB4A |  |  | 1928 | 0.049 | -0.1254 | No |
| 9 | LIFR |  |  | 1972 | 0.048 | -0.1254 | No |
| 10 | CDC14B |  |  | 2270 | 0.040 | -0.1675 | No |
| 11 | CDK6 |  |  | 2322 | 0.039 | -0.1700 | No |
| 12 | PDLIM5 |  |  | 2517 | 0.034 | -0.1963 | No |
| 13 | HMGCS1 |  |  | 2674 | 0.031 | -0.2169 | No |
| 14 | BMPR1B |  |  | 2711 | 0.030 | -0.2182 | No |
| 15 | IDI1 |  |  | 2749 | 0.030 | -0.2199 | No |
| 16 | SLC26A2 |  |  | 2805 | 0.028 | -0.2246 | No |
| 17 | TPD52 |  |  | 2901 | 0.027 | -0.2360 | No |
| 18 | STK39 |  |  | 2943 | 0.026 | -0.2389 | No |
| 19 | SRP19 |  |  | 3006 | 0.025 | -0.2453 | No |
| 20 | PGM3 |  |  | 3839 | -0.001 | -0.3792 | No |
| 21 | SEC24D |  |  | 3914 | -0.005 | -0.3905 | No |
| 22 | DNAJB9 |  |  | 3929 | -0.006 | -0.3919 | No |
| 23 | DBI |  |  | 4122 | -0.017 | -0.4204 | No |
| 24 | VAPA |  |  | 4263 | -0.026 | -0.4392 | No |
| 25 | UAP1 |  |  | 4281 | -0.027 | -0.4379 | No |
| 26 | GNAI3 |  |  | 4426 | -0.037 | -0.4557 | No |
| 27 | XRCC6 |  |  | 4437 | -0.039 | -0.4516 | No |
| 28 | AKT1 |  |  | 4544 | -0.047 | -0.4618 | No |
| 29 | CCND3 |  |  | 4578 | -0.050 | -0.4599 | No |
| 30 | ANKH |  |  | 4600 | -0.051 | -0.4558 | No |
| 31 | ABCC4 |  |  | 4628 | -0.054 | -0.4523 | No |
| 32 | ADRM1 |  |  | 4687 | -0.059 | -0.4531 | No |
| 33 | SCD |  |  | 4717 | -0.061 | -0.4488 | No |
| 34 | RPS6KA3 |  |  | 4757 | -0.065 | -0.4456 | No |
| 35 | NDRG1 |  |  | 4809 | -0.068 | -0.4439 | No |
| 36 | FKBP5 |  |  | 5047 | -0.087 | -0.4694 | Yes |
| 37 | SLC38A2 |  |  | 5128 | -0.094 | -0.4686 | Yes |
| 38 | B4GALT1 |  |  | 5170 | -0.099 | -0.4608 | Yes |
| 39 | HOMER2 |  |  | 5194 | -0.101 | -0.4497 | Yes |
| 40 | ELOVL5 |  |  | 5226 | -0.103 | -0.4396 | Yes |
| 41 | ABHD2 |  |  | 5255 | -0.107 | -0.4286 | Yes |
| 42 | UBE2I |  |  | 5293 | -0.109 | -0.4186 | Yes |
| 43 | TMEM50A |  |  | 5305 | -0.110 | -0.4044 | Yes |
| 44 | ITGAV |  |  | 5309 | -0.110 | -0.3888 | Yes |
| 45 | FADS1 |  |  | 5324 | -0.111 | -0.3748 | Yes |
| 46 | IQGAP2 |  |  | 5451 | -0.125 | -0.3769 | Yes |
| 47 | ACTN1 |  |  | 5765 | -0.176 | -0.4017 | Yes |
| 48 | B2M |  |  | 5823 | -0.188 | -0.3834 | Yes |
| 49 | PA2G4 |  |  | 5841 | -0.193 | -0.3580 | Yes |
| 50 | HMGCR |  |  | 5906 | -0.208 | -0.3378 | Yes |
| 51 | XRCC5 |  |  | 5923 | -0.213 | -0.3093 | Yes |
| 52 | SMS |  |  | 5940 | -0.218 | -0.2800 | Yes |
| 53 | DHCR24 |  |  | 5943 | -0.219 | -0.2484 | Yes |
| 54 | SPCS3 |  |  | 5948 | -0.220 | -0.2169 | Yes |
| 55 | AKAP12 |  |  | 5988 | -0.234 | -0.1890 | Yes |
| 56 | PMEPA1 |  |  | 6009 | -0.241 | -0.1571 | Yes |
| 57 | ACSL3 |  |  | 6078 | -0.284 | -0.1265 | Yes |
| 58 | CENPN |  |  | 6090 | -0.291 | -0.0858 | Yes |
| 59 | LMAN1 |  |  | 6130 | -0.322 | -0.0450 | Yes |
| 60 | CCND1 |  |  | 6203 | -0.454 | 0.0097 | Yes |
Table: GSEA details [plain text format]

  

Fig 2: HALLMARK\_ANDROGEN\_RESPONSE: Random ES distribution      
 Gene set null distribution of ES for **HALLMARK\_ANDROGEN\_RESPONSE**
